# How to find the right drug for each patient? Advances and challenges in pharmacogenomics

**DOI:** 10.1016/j.coisb.2018.07.001

**Published:** 2018-08

**Authors:** Angeliki Kalamara, Luis Tobalina, Julio Saez-Rodriguez

**Affiliations:** 1 RWTH Aachen University, Faculty of Medicine, Joint Research Centre for Computational Biomedicine, Aachen, Germany; 2 European Molecular Biology Laboratory, European Bioinformatics Institute, Wellcome Trust Genome Campus, Hinxton, UK; 3 Heidelberg University, Faculty of Medicine, Institute of Computational Biomedicine, Heidelberg, Germany

## Abstract

Cancer is a highly heterogeneous disease with complex underlying biology. For these reasons, effective cancer treatment is still a challenge. Nowadays, it is clear that a cancer therapy that fits all the cases cannot be found, and as a result the design of therapies tailored to the patient's molecular characteristics is needed. Pharmacogenomics aims to study the relationship between an individual's genotype and drug response. Scientists use different biological models, ranging from cell lines to mouse models, as proxies for patients for preclinical and translational studies. The rapid development of "-omics" technologies is increasing the amount of features that can be measured in these models, expanding the possibilities of finding predictive biomarkers of drug response. Finding these relationships requires diverse computational approaches ranging from machine learning to dynamic modeling. Despite major advances, we are still far from being able to precisely predict drug efficacy in cancer models, let alone directly on patients. We believe that the new experimental techniques and computational approaches covered in this review will bring us closer to this goal.

## Highlights

• Cancer pharmacogenomics holds the promise to find biomarkers to improve patient treatment and stratification in cancer.
• We summarize biological cancer models and computational approaches for cancer pharmacogenomics studies.
• Translation of laboratory finding to the clinic is still an open challenge.

## Pharmacogenomics and personalized medicine in cancer

**Figure 1:**
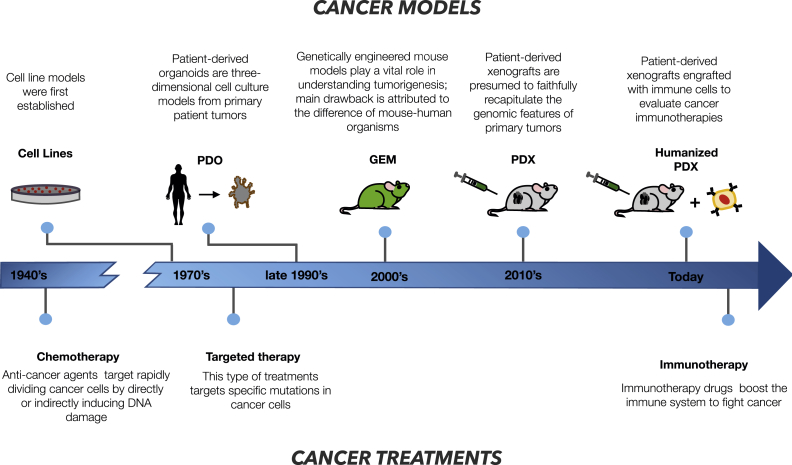
**Timeline of developments towards personalized medicine**. The figure shows schematically the timeline of development of cancer models and cancer therapies to address the challenges in personalized medicine. Dates are orientative as of when these developments started to be widespread.

Cancer is a molecularly heterogeneous disease with complex biology and one of the major causes of death worldwide. Common treatment options range from surgical intervention to radiation and drug therapy. Non-targeted chemotherapeutic agents have been the mainstay of conventional drug treatment. Targeted therapies constitute more advanced treatment options that aim to target specific molecular changes in cancer cells, typically based on kinase inhibitors and monoclonal antibodies. Immunotherapies that potentiate the response of the immune system against tumors [1] are a recent addition to the arsenal of treatment options, particularly promising in combination with targeted therapies [2] (Figure 1).

Regardless of the treatment, while some patients respond to it, a great percentage of them exhibit or develop resistance. Resistance to cancer therapy is largely attributed to inter-patient heterogeneity (between patients of the same tumor type) [3] as well as intra-tumor heterogeneity (between different parts of the tumor within a patient) [4]. Which treatment is likely to suit a patient depends on patient-specific molecular characteristics and clinical information. A well-known example is the treatment of cancers harboring a BRAF mutation with Vemurafenib, an inhibitor designed to target specifically the mutated form of BRAF. Melanoma patients harbouring a BRAF mutation often respond initially to BRAF inhibitor treatment, but typically resistance develops. The appearance of the resistance can be delayed with the combination of BRAF and MEK inhibitors [5]. However, colon cancer patients harboring the same mutation typically exhibit resistance to Vemurafenib [6]. While there are some such cases of established molecular biomarkers for specific drugs, these are scarce and as a consequence few cancer patients benefit fully today from precision oncology [7].

Pharmacogenomics attempts to address the issue of finding the appropriate biological markers by understanding how individuals' genetic variants influence drug response to treatment. A broad characterization of cancer cells is important to understand drug efficacy. In particular, the effectiveness of a targeted therapy may depend on more factors than the presence of the alteration targeted by the drug itself. For instance, the failure of Vemurafenib treatment in colon cancer is related to a feedback activation of EGFR, which does not happen in melanoma cells because these exhibit a low expression of this receptor [8], although eventual EGFR activation may also happen in melanoma cells, conferring them resistance to BRAF inhibitors [9]. This knowledge can aid in the discovery of new solutions. For example, in the above case it led to the combination of an EGFR inhibitor such as Erlotinib with Vemurafenib for improved treatment response of colon patients [6]. Such drug combinations are intensively studied to improve treatment and in particular to overcome resistance to monotherapy 10, 11, 12.

Here, we review some of the recent experimental and computational developments in the field of pharmacogenomics and personalized medicine in cancer.

## Experimental models of cancer therapeutics

Ideally, the data for a pharmacogenomic study would be generated from patients, since we desire to predict on them the effect of different treatments. As this is not feasible, we need to rely on experimental systems that present similar characteristics to the primary tumor but can be manipulated easily in the laboratory (Figure 1).

Cancer cell line *in vitro* models are the most popular amongst all models in personalized oncology [13]. Since they can be relatively easily grown in the laboratory, they can be subject to multiple experiments, including deep molecular characterization as well as treatment with many drugs, drug combinations included. Connection of molecular characterization and treatment provide a rich substrate to study pharmacogenomic associations ∗14, 15, 16. Nevertheless, cell line models present important limitations. A major drawback is the lack of tumor microenvironment and of intrinsic heterogeneity compared to the original tumor.

Patient-derived organoids (PDO), three-dimensional cell cultures derived from a patient's tumor 17, 18, ∗∗19, are considered to be a better *in vitro* model. They represent more accurately the intrinsic environment of the primary tissue as they can include multiple cell types and self-organize into tissue-like structures. Furthermore, drug response in organoids seems to associate with genomic alterations more similarly to real tumors than cell lines [20]. However, a main drawback of PDO models is the difficulty to maintain them in long-term cultures. Furthermore, organoids typically lack immune cells and hence cannot be used to study the outcome of a treatment designed to interact with the immune system.

Mouse-based *in vivo* models are a valuable yet expensive tool for preclinical evaluation of novel therapeutic strategies in cancer. Patient-derived tumor xenograft (PDX) models 21, 22, 23 are obtained by direct implants of patient's tumor cells or tissue fragments in immunodeficient mice and can recapitulate the heterogeneity and intrinsic drug sensitivity of the primary tumor [24]. However, they are a limited model of tumors *in vivo* and in particular of the interaction of the tumor with the immune system. A genetically engineered mouse (GEM) model [25], in turn, is a mouse whose genome has been modified by genetic engineering techniques to initiate tumorigenesis. Even though GEM models do not reflect the complex heterogeneity of a human tumor, they harbor significant genetic heterogeneity [26]. Still, the difference between mouse and human organism is the great obstacle for immuno-oncology drug discovery studies. This gap is filled by “humanized” PDX models which are additionally engrafted with human immune cells to imitate the human tumor microenvironment. Humanized PDX models have been developed to study immunotherapies in melanoma and hepatocellular carcinoma providing results similar to clinical outcomes 27, 28.

Finally, *exvivo* models involve taking a sample out of the organism or patient and studying it under more controlled conditions than *in vivo*. The samples are not cultured for long periods, so that they still retain the characteristics of the original donor. This approach has been applied, for instance, to the study of patient specific drug response in blood cancers ∗29, 30. The necessary amount of material for these studies may limit its application, specially for solid tumors. However, recent developments such as refined readouts that measure early events as proxies of later drug efficacy [31], and microfluidic technologies that require small amounts of samples and reagents, will facilitate drug screening in human biopsies, including drug combination studies 32, 33.

## Data collection and drug screening

**Figure 2:**
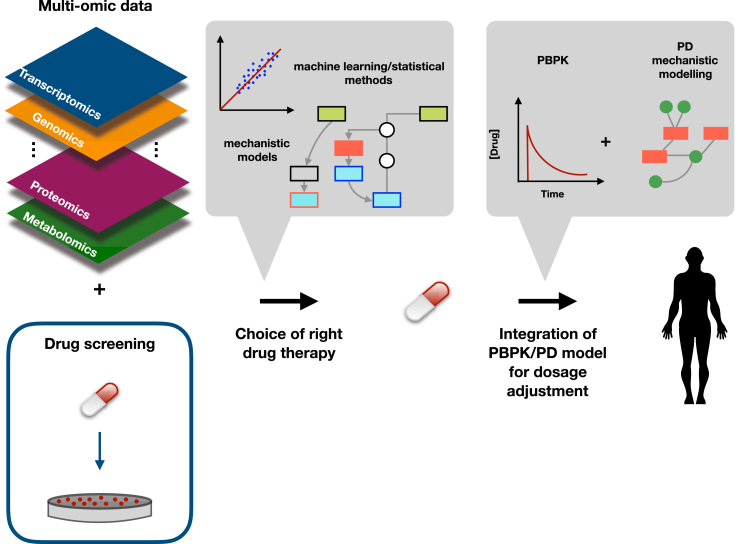
**Linking pharmacogenomics to pharmacokinetics for precision oncology**. Pharmacogenomics can provide appropriate drug candidates to target at the molecular level the tumor. Integrating this pharmacodynamic (PD) information into a physiology-based pharmacokinetic (PBPK) model would allow us to precisely define the best drug dosage for individual patients.

To identify biomarkers, the cancer models should be analysed as thoroughly as possible (Figure 2). To date, large-scale next-generation sequencing-based (genomic, epigenomic and transcriptomic) data are available in particular for cell lines ∗14, 34. Mass spectometry-based data (e.g. proteomic 35, 36, 37 and metabolomic [38]) are less common but increasing their coverage. While they have already provided useful insights, by for instance describing how genomic alterations propagate to the proteomic layer [36], the role of protein-complex regulation for detecting cell vulnerabilities [39], the importance of metabolism in drug resistance [40] or identifying glutathione biosynthesis as a potential metabolic vulnerability of PI3K pathway altered breast cancers cells [41], we look forward to larger-scale collections as a means to find novel protein or metabolite biomarkers. In addition, development of new biological techniques will foster new discoveries, like the improvement of platforms for annotating genomic aberrations that lead to the identification of previously uncharacterized potential driver mutations [42]. An additional challenge involves the data quality (e.g noise, technical variation, difference in platforms used to generate measurements) which can interfere with the interpretation of the underlying biology.

Cell line drug screening is the process by which anti-cancer compounds are screened against a panel of cell lines and measure the *in vitro* drug response. When screening a single agent, standard metrics to measure drug response are the half-maximal inhibitory concentration (IC_50_) as a measure of drug potency, the area under the dose–response curve (AUC) that takes into account drug potency and efficacy and the newly introduced GR_50_ half-maximal growth rate inhibition, based on the comparison of growth rates in the presence and absence of drug (Box 1). When screening combinations of agents the focus is on metrics that assess synergy, such as the Loewe additivity model or the Bliss independence model [43] (Box 1).

Box 1 **Selected mathematical and computational terms in pharmacogenomics**

**Assessing drug activity**

IC_50_ is the half-maximal inhibitory concentration and is a measure of potency of a compound inhibiting a specific target. Denotes the concentration of the inhibitor where the response (cell counts) drops in half.

GR_50_ is the half-maximal growth rate inhibition, based on the comparison of growth rates in the presence and absence of drug. GR_50_ quantifies the potency of a drug on a per-division basis to correct for differences in scoring for differently growing cells [51].

AUC is the area under the dose–response curve and combines potency and efficacy of a drug.

**Assessing drug synergy**

Drug combinations exploit the chances for better efficacy, decreased toxicity and reduced development of drug resistance. A drug combination is usually classified as synergistic or antagonistic, depending on the deviation of the observed combination response from the expected effect calculated based on a reference model of non-interaction. The most widely used models to assess drug combinations are the Loewe additivity and Bliss independence models.

Loewe additivity model requires information on the dose–response relationship of the individual drugs and assumes that the combination of two drugs that do not interact would give the same result as combining one drug with itself [43].

Bliss independence model assumes that drugs act independently from each other, through different mechanisms of action, but both contributing to a common result. Its greatest limitation lies in the assumption of known mechanism of action of drugs involved which is often not feasible when new drugs are studied [43].

**Testing statistical drug associations**

Analysis of Variance (ANOVA) is a statistical model used to determine whether there are any statistically significant differences between multiple independent (unrelated) groups, as a generalization of the t-test. In the context of drug associations it can be used to identify significant associations between groups of cell lines, each of which defined by certain features or genomic alterations, and drugs [14].

**Drug sensitivity prediction models**

Different statistical or machine learning models can be built to predict the response of cell lines based on the features of the cell lines, typically genome-wide molecular characterization.

Linear models are a simple and common choice to predict drug response in cell lines. The drug responses are modelled as a continuous vector *y* which is assumed to depend on the linear combination of the features ***X*** and an unknown vector *β so as*

Y∼Xβ

Where Y is a vector of the drug response variable (dependent variable) e.g IC_50_/AUC/GR_50_, X∈ ${\mathbb{R}}^{N\times M}$ a matrix consists of predictors where *N* denotes the number of genes and *M* the number of samples e.g gene expression matrix of the cell lines and *β* is the coefficients vector that the linear model needs to learn for future predictions.

An advantage of these models is their ability to identify predictive genes of drug response and are easy to interpret. In molecular data, since the number of genes is much greater than the number of samples, introducing a regularization term to penalize the complexity of the model is a common strategy to control overfitting, resulting in the ridge, lasso or elastic net regularizations.

Non-linear models

Non-linear regression models have been used for drug response prediction on cancer cell lines. These models tend to have better overall accuracy compared to regularized linear approaches, however their interpretability is not as straightforward. Kernel-based methods (e.g support vector machines, artificial neural networks, Bayesian kernel-based multi-task learning [57]) and ensemble methods, e.g random forests, are some examples. Support Vector Machines perform linear regression on a higher dimensional space defined by the chosen kernel, leading to non-linear regression on the original feature space. Neural networks are able to find the appropriate non-linear transformation of the input data by using a layered composition of basic non-linear transformations (e.g. sigmoid function), provided enough data is available to estimate all the parameters involved. Random forests work by generating multiple regression trees whose average prediction outperforms the prediction of any single tree alone.

Alt-text: Box 1

The NCI60 panel [44], which utilizes 60 cancer cell lines, is the pioneer in the cancer cell line screening and aims to identify and characterize novel compounds with tumor-killing properties. These cell lines were molecularly characterized to identify biomarkers of response, providing thus the first resource for cancer pharmacogenomics. While a powerful resource, the relatively low number of cell lines limit the ability to discern biomarkers. This is particularly hampered by the strong association between tissue type and genomic and transcriptomic alterations, that can not be dissected with only 60 cell lines for 9 tissue types. For this reason, more recently, pharmacogenomics screens were performed with an order of magnitude more cell lines, such as the Cancer Therapeutic Response Portal (CTRPv2) [15], Cancer Cell Line Encyclopedia (CCLE) [34], Genomics in Drug Sensitivity in Cancer (GDSC) [14] and Genentech Cancer Cell Line Screening Initiative (gCSI) [16]. These large-scale studies are partially overlapping, though their results do not fully agree, and it is still an open debate if and how they can be used together ∗14, 16, 45, 46, 47, 48, 49, 50. In addition, the standard metrics used to characterize drug response (IC_50_ and AUC) have also been scrutinized, and refinements that take into account confounding factors such as the proliferation rate of the different cells, might improve results and comparability across studies [51]. These efforts have focussed so far on single agents, and the field is now gearing towards combinations of drugs. First combination screenings are becoming available 52, 53 and more are likely to come soon, enabling us to find biomarkers for combination therapies to select the most effective additional drug to improve therapeutic outcome in cases of drug resistance resulting from targeting a single gene.

While we currently have a rich molecular characterization of the samples, the characterization of the phenotypic effect of the drugs is comparatively very simple, generally represented by their cytotoxic effect (e.g. IC_50_). A richer output, such as cell morphology, motility or molecular changes, would provide more insights into the effect of the drug, thereby helping to better understand and predict drug activity [54]. Advances in expression profiling [55] and microscopy technologies [56] allow a high-throughput characterization [20] of richer phenotypes that is starting to be applied at large scale.

## Computational approaches in cancer research

The amount of data generated by these large-scale studies has led to the development of computational methods for understanding and predicting the relationship between drugs and genes. The simplest mean to discover pharmacogenomic associations is using statistical tests such as the Analysis of Variance (ANOVA) (Box 1), which can be used to identify, for instance, whether oncogenic alterations present in patients are markers of differential drug sensitivity in cell lines ∗14, 15, 34.

To go beyond the drug association (which markers render cell lines sensitive/resistant to a given drug) to actual prediction of drug response, various statistical and machine learning approaches can be used, ranging from linear regression models to non-linear models such as kernel methods, neural networks, random forests and support vector machines [57] (Box 1).

A main obstacle that these methods face is the large number of input features versus the low number of samples, e.g in a typical gene expression experiment the number of features (genes) outnumbers the sample size. One way to combat the high-dimensionality issue is by using dimensionality (feature) reduction methods, either in a data-driven way or using prior knowledge on pathways and other molecular processes to derive mechanistic features such as protein and pathway activities ∗57, 58, 59, 60. These features can be directly associated to drug response to, for example, identify transcription factor activities as biomarkers of drug response [59], or as input for machine learning algorithms [57].

A complementary strategy to tackle the low amount of samples problem is to use multi-task learning algorithms 61, 62, in which instead of learning prediction models for one drug at a time, a model for all the drugs is jointly learnt, harvesting information available from all the samples at once. Here, information on the chemical features of the compounds can be used as features into the models, as complementary knowledge to the molecular characterization on the cell lines 63, 64. One collaborative competition of the DREAM challenges investigated the best strategies to predict drug response from "-omics" (transcriptomics, genomics, methylation, and protein data) in breast cancer cell lines [57]. The best performance was achieved using bayesian multi-task multiple kernel learning approach and transcriptomics was the most predictive amongst the "-omic" data.

Despite advances, existent computational models suffer from low predictive power and poor generalization, as can be noted when checking the performance of methods published, also among the best methods in the DREAM challenge [57]. There are multiple possible reasons for this, including (i) noise in the data, and the aforementioned (ii) relative low number of samples when compared to the features, (iii) incomplete omics characterization, in particular in terms of proteomic and metabolomics, and (iv) limited readouts. Noise in the data can be either biological or technical and while methods to correct for technical variation are developed allowing us to bring datasets of different platforms together, the discussion on what is the correct way to apply them is not settled, adding uncertainty to downstream results [65]. The low amount of samples toughens the discrimination of the true signal from noise. Incomplete “omics” characterization leaves open the possibility that the answer to our questions lies in the things that have not been measured. Finally, the number of readouts is very relevant as subsequently the data used for models is vastly static (prior to treatment), while the effect of the drug is a dynamic process, whereby the drug modulates molecular components of the cell, that responds to this as an integrated system, as the target of the drug is often embedded in a complex molecular network that includes multiple pathways, crosstalks among them, and feedbacks.

A different approach that can help to shed lights on these dynamic processes that underlie drug efficacy involves using mechanistic dynamic models. These models aim to understand deregulation of signal transduction in disease and can be used to predict the effect of a drug. The models can be built from prior knowledge and/or experimental data. The data can be the basal, although dynamic data upon perturbation provides additional information on the functionality of the underlying pathways. Logic formalisms for their simplicity can capture the multiple pathways that are involved in drug response and resistance mechanism, and they have been effectively used to predict drug combinations 66, 67, 68. These models do not only predict treatments [66] or reveal sensitivity markers not related to any known genomic biomarker [67], but also provide insight on the mechanism that underpin drug efficacy. Models that describe the biochemical reactions are more detailed and therefore do not scale up so well, although recent advances in simulation and parameter estimation makes it possible to use them for large networks and to predict drug response [69]. Finally, data-driven models applied to perturbation data can also uncover combination therapies [70].

## Summary and outlook

Personalized (precision) medicine aims to match the best treatment to each patient. In this review, we discussed the importance of pharmacogenomics for this goal in oncology. We have reviewed the different experimental and computational approaches used in preclinical studies. In particular, the growth of multi-omic data combined with the measurements from large-scale drug screening studies provides a rich substrate to uncover predictive biomarkers that span from single-gene markers to signatures of processes and pathways. So far, efforts to validate prognostic gene signatures have typically used existing public datasets rather than with newly collected patient samples. An example of such validation was provided by Ref. [71] who validated predictive molecular parameters suggested in Ref. [72] in an independent retrospective cohort of AML patients to predict overall survival at 3 years. These molecular parameters were derived from a comprehensive collection of patients' genomic data matched with clinical outcome and included coding exons from 111 myeloid cancer related genes and cytogenetic profiles from 1540 leukemia patients who underwent intensive treatment within 3 different clinical trials. Translating predictive biomarkers systematically in the clinic today remains a great challenge. Even if data generated were not suffering from technical biases, intra-tumor heterogeneity and tumor evolution are major thorns. For instance, sequencing a specific part of a biopsy could result in a significantly different outcome than any other part of it and as a result a treatment based on the first one would have limited benefit on patients.

**Figure 3:**
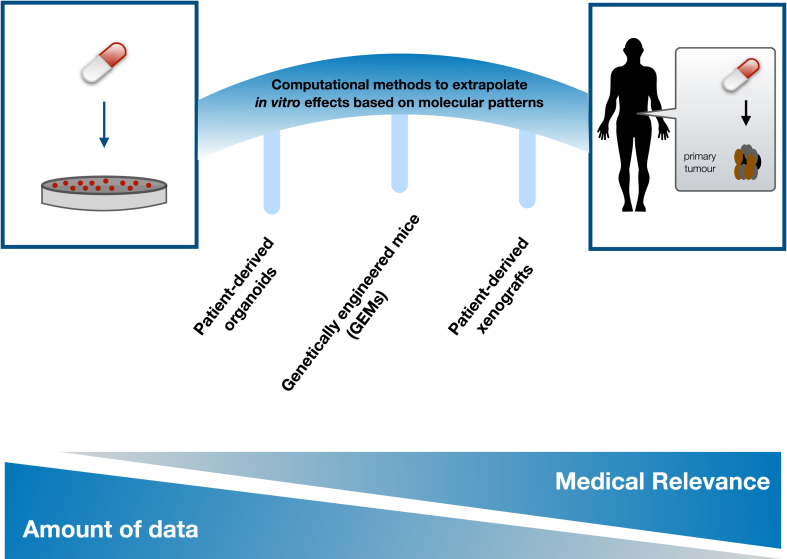
**From preclinical studies to the clinic**. To bridge the formidable gap between *in vitro* pre-clinical data and patients, computational models can help to extrapolate from the former to the latter. To support this bridge, data from the different preclinical models can be integrated. The different models available have a tradeoff between their ease of use (and thus amount of data available) and medical relevance (defined by how close they are to a real patient).

In addition, while clinically approved biomarkers can be recapitulated in cell line screenings [14], not many biomarkers from *in vitro* screenings have been successfully translated into the clinic. Efforts to predict *in vivo* drug sensitivity using human cancer cell lines across diverse tumor types via building machine learning models [73] have been reported, but they remain to be systematically validated. A major bottleneck is the limited amount of available data of clinical trials where molecular information before treatment is coupled to therapeutic outcome. We believe that this can be partially compensated by data from other cancer models, integrating the different data via computational approaches (Figure 3).

Also, new considerations for the design of prospective clinical trials could benefit future research studies. For instance, depending on the cancer type considered in the trial, sample from patients' could be collected upon their enrollment. Then, even if the trial fails, the collected samples could be exploited to shed light on the different levels of response observed. Given the decreasing cost of sequencing technologies (or the currently outmoded microarray technology) analysis of these samples could be notably beneficial.

Most analyses so far have focused on single agents. Analysis of combinations focuses on finding synergy, although their effect on a patient population can be due to the distinct effect of independent drugs on different subpopulations [74]. Furthermore, combinations studied so far have used drugs targeting intracellular processes 11, 52, 53 and therapeutic modalities such as immunotherapies open new opportunities for the treatment of patients but also require novel approaches to test and analyze them [10].

Pharmacogenomics can tell us which molecular alterations characterize a cell line, and eventually a tumor in a patient, more likely to respond to a drug. However, this alone will not guarantee a successful outcome in the clinical setting, as the drug needs to reach the tumor with the right concentration for the right duration that will lead to tumor shrinkage. These questions are the focus of the field of pharmacokinetics (PK). In particular, physiologically-based pharmacokinetic (PBPK) [75] models take into account the individual's biometrics and physiological characteristics (often including genetic variants) to study the absorption, distribution, metabolism and excretion of a drug in the body and consequently predict the concentration of the drug that reaches the target tumor tissue. This is typically combined with a pharmacodynamic model (PD) that describes the effect of the drug on the target cell. To date only compartmental models [76] have been established and applied in oncology. A PD model, however, could be informed by the pharmacogenomics one to build a PBPK/mechanistic-PD model to predict the actual tumor growth over time given the initial dose that is adjusted to a specific cancer patient (Figure 2). Developing such models is very challenging due to the large amount of data and diverse knowledge required, but could be a decisive tools towards personalized oncology. Finally, genome editing tools may also be a possible treatment in the future, in particular applying the CRISPR-Cas9 technology [77].

As we have seen, the current experimental and computational developments are fostering new advances in pharmacogenomics. However, the translation of the findings into clinical practice is still lagging behind. We expect to see a greater emphasis on research addressing this challenge in the following years.

## Conflict of interest

None declared.
